# Long non‐coding RNA MALAT1 mediates hypoxia‐induced pro‐survival autophagy of endometrial stromal cells in endometriosis

**DOI:** 10.1111/jcmm.13947

**Published:** 2018-10-15

**Authors:** Hengwei Liu, Zhibing Zhang, Wenqian Xiong, Ling Zhang, Yu Du, Yi Liu, Xingao Xiong

**Affiliations:** ^1^ Department of Obstetrics and Gynecology Tongji Medical College Union Hospital Huazhong University of Science and Technology Wuhan China; ^2^ Department of Physiology Wayne State University Detroit Michigan; ^3^ Department of Obstetrics and Gynecology Wayne State University Detroit Michigan; ^4^ Department of Ear‐Nose‐Throat (ENT) Tongji Medical College Union Hospital Huazhong University of Science and Technology Wuhan China

**Keywords:** apoptosis, autophagy, endometriosis, HIF‐1α, hypoxia, lncRNA‐MALAT1

## Abstract

Endometriosis is a common gynecological disease characterized by diminished apoptosis, sustained ectopic survival of dysfunctional endometrial cells. Hypoxia has been implicated as a crucial microenvironmental factor that contributes to endometriosis. It has been reported that long non‐coding RNA MALAT1 (lncRNA‐MALAT1) highly expressed in endometriosis and up‐regulated by hypoxia. Hypoxia may also induce autophagy, which might act as cell protective mechanism. However, the relationship between lncRNA‐MALAT1 and autophagy under hypoxia conditions in endometriosis remains unknown. In the present study, we found that both lncRNA‐MALAT1 and autophagy level were up‐regulated in ectopic endometrium from patients with endometriosis, and its expression level correlates positively with that of hypoxia‐inducible factor‐1α (HIF‐1α). In cultured human endometrial stromal cells, both lncRNA‐MALAT1 and autophagy were induced by hypoxia in a time‐dependent manner and lncRNA‐MALAT1 up‐regulation was dependent on HIF‐1α signalling. Our analyses also show that knockdown of lncRNA‐MALAT1 suppressed hypoxia induced autophagy. Furthermore, inhibiting autophagy with specific inhibitor 3‐Methyladenine (3‐MA) and Beclin1 siRNA enhanced apoptosis of human endometrial stromal cells under hypoxia condition. Collectively, our findings identify that lncRNA‐MALAT1 mediates hypoxia‐induced pro‐survival autophagy of endometrial stromal cells in endometriosis.

## 1 INTRODUCTION

Endometriosis is a common, complex benign gynecological disorder, defined as the extra‐uterine growth of endometrial tissues.^1^ It is one of the most common causes of chronic pelvic pain and reduced fertility, and it affects about 10%‐15% of women of child‐bearing age. It is widely accepted that spontaneous programmed cell death of endometrial cells is impaired in women with ovarian endometriosis, and this reduced susceptibility to apoptosis might permit the abnormal survival of endometrial cells in ectopic locations.^2^, ^3^ Nevertheless, the mechanism underlying the reduced endometrial cell apoptosis in endometriosis remain largely unknown.

Hypoxia has a pathophysiological effect through the process of disease and regulation of gene expression.^4^ Numerous studies have suggested that peritoneal hypoxia is intricately involved in the pathogenesis of ovarian endometriosis.^5^, ^6^ The key factor mediating cellular hypoxia response is the hypoxia‐inducible factor‐1 (HIF‐1), which is a heterodimeric transcription factor composed of an O_2_‐sensitive α‐subunit and a constitutively expressed β‐subunit.^7^ In women susceptible to endometriosis, it is probable that retrograde menstruation of endometrial cells or debris that attach to the peritoneal wall may be exposed to hypoxic‐ischaemic peritoneal microenvironment.^8^ Previous studies reported that hypoxia may play a role in the survival and angiogenesis of retrograde endometrial cells in implanted ectopic endometriotic lesions.^9^ Moreover, during this process HIF‐1α regulate the expression of multiple target genes involved in different processes including angiogenesis, apoptosis, and autophagy.^10^, ^11^

Autophagy is an evolutionarily conserved cellular catabolic process by which cells capture bulk intracellular components like abnormal protein aggregates and damaged organelles and degraded them through delivery to lysosomes.^12^ Autophagy is vital in a range of pathophysiological situations, and dysregulated autophagy is associated with various human diseases, including neurodegenerative diseases, infection, and malignancy.^13^ It is generally believed that autophagy plays a crucial role in protecting cells to survival by preventing apoptosis under various metabolic stresses microenvironment, such as hypoxia or oxidative stress.^14^, ^15^, ^16^ Interestingly, our previous studies as well as others revealed that autophagic process was up‐regulated in ovarian endometriomas.^17^, ^18^ However, despite the expansion of our knowledge about autophagy, the mechanisms responsible for the aberrant activation of autophagy and the detailed effect of autophagy on survival of human endometrial cells under the hypoxia condition remain largely unknown in the context of endometriosis.

Long non‐coding RNAs (lncRNAs) are a family of RNAs longer than 200 nucleotides with limited or no protein‐coding potential.^19^ An increasing number of lncRNAs have been reported to participate in various biological processes such as cell differentiation, apoptosis, and autophagy and are involved in the pathogenesis of endometriosis.^20^, ^21^ Among them, lncRNA metastasis‐associated lung adenocarcinoma transcript 1 (lncRNA‐MALAT1), which was first described to be associated with metastasis of lung tumours, is highly conserved in mammalian species.^22^ Researchers found lncRNA‐MALAT1 was robustly induced by hypoxia in several cancer cell types.^23^, ^24^, ^25^ In addition, several studies reported that lncRNA‐MALAT1 modulates the activity of autophagy.^26^, ^27^ More importantly, recent research indicated expression of lncRNA‐MALAT1 was significantly increased in the ectopic endometrium of patients with ovarian endometriosis.^28^ However, the correlation between lncRNA‐MALAT1 and autophagy remains unclear in the context of endometriosis. Therefore, considering the fact that the elevated expression of lncRNA‐MALAT1 in ovarian endometriosis and its connection with autophagy as well as the up‐regulation of both lncRNA‐MALAT1 and autophagy were up‐regulated by hypoxia, we have been suggested that up‐regulation of lncRNA‐MALAT1 by hypoxia may promote autophagy which acts as a cell pro‐survival mechanism in endometriosis.

## 2 MATERIALS AND METHODS

### 2.1 Institutional ethical approved and informed consent

All tissue samples were collected according to the protocols that were approved by the local Ethics Committee of Tongji Medical College, Huazhong University of Science (IORG No: IORG0003571) and the research was carried out according to the World Medical Association Declaration of Helsinki. Written informed consent was obtained from every participant before tissue samples collection.

### 2.2 Patients and tissue collection

Tissue specimens were acquired during 2014‐2016 at the Department of Obstetrics and Gynecology, Union Hospital, Tongji Medical College, Huazhong University of Science and Technology from 106 non‐pregnant women of childbearing age (23‐45 years). The recruited patients had regular menstrual cycles (between 26 and 32 days) and no subject received any pre‐operative hormone treatments within the 3 months. All surgeries were performed during the proliferative stage of the patients’ menstrual cycle, which was confirmed based on clinical or histologic criteria.

From the healthy control group an endometrial specimen (n = 30) was collected from healthy fertile women who were undergoing laparoscopic tubal ligation or reversal of tubal sterilization by hysteroscopy. The matched eutopic endometrium (n = 30) was collected from the same patient with ovarian endometriosis (n = 30) undergoing laparoscopic treatment for infertility and/or ovarian cysts. Ovarian endometriotic tissues (cyst diameter measured by ultrasound ranged from 38 to 72 mm) were carefully stripped from the inner cyst wall avoiding contamination with ovarian tissues. In the endometriosis group, all patients showed stage III or IV endometriosis according to the American Society for Reproductive Medicine classification.^29^ All specimens were immediately frozen and stored in liquid nitrogen for RNA and protein extraction or paraffin embedded for immunohistochemistry; they were all analyzed at the same time. The clinical data of the patients are summarized in Table S1.

### 2.3 Immunohistochemistry

Immunohistochemical staining was performed as described in our previous study. Formalin‐fixed paraffin‐embedded specimens of normal, eutopic, and ectopic endometrium were cut into 5 μm sections. Then sections were deparaffinized and antigen‐retrieval was performed in sodium‐citrate buffer (10 m mol L^−1^, pH 6) for 10 minutes at 90°C. Endogenous non‐specific peroxidase activity was quenched by treatment with 3% of hydrogen peroxide for 30 minutes at room temperature (RT). Then followed by 30 minutes incubation with 1% bovine serum albumin to block non‐specific binding. Tissue sections were then incubated with the primary anti‐HIF‐1α (diluted 1:150; Affinity, USA) and microtubule‐associated proteins 1A/1B light chain 3 (LC3) (diluted 1:100; Abcam, Cambridge, UK) overnight at 4°C. Sections were subsequently washed with phosphate‐buffered saline solution (PBS) and incubated with peroxidase‐labelled goat anti‐rabbit IgG (diluted 1:500; Servicebio Biotech, Wuhan, China) for 30 minutes at RT. 3,30‐Diaminobenzidine tetrahydrochloride substrate (DAB) (Beyotime, Wuhan, China) was used as a substrate and sections were lightly counterstained with hematoxylin, dehydrated, and mounted. Negative control sections were incubated with an isotype‐matched anti‐rabbit antibody. Characteristics of antibody used for immunohistochemistry are listed in Table S3.

Scoring of protein expression was performed according to the intensity of staining and the percentage of positive cells. The intensity of staining was graded as 0 = no staining, 1 = weak staining, 2 = moderate staining, and 3 = strong staining. The percentage of stained cells was graded as following; 0 = no staining, 1 ≤ 10%, 2 = 11%‐50%, 3 = 51%‐80%, and 4 ≥ 81%. The final score was calculated by multiplying the two scores. Scoring was done blindly and by two independent observers.

### 2.4 Isolation and culture of primary human endometrial stromal cells

We purified primary human endometrial stromal cells as described previously with slight modification.^18^ Briefly, washed endometrium tissues were minced into 1‐ to 2‐mm pieces with a sterile surgical scissors and digested in PBS containing 2 mg/mL of type II collagenase (0.1%, Sigma‐Aldrich, USA) for 45‐60 minutes at 37°C with constant agitation. The resulting suspension was filtrated through sterile 150 and 37.4 μm sieves in turn to remove undigested epithelial cells and debris. The filtrate was then centrifuged at 1000 *g* for 5 minutes and then further cultured in Red Blood Cell Lysis Buffer for 10 minutes to remove erythrocytes. After being centrifuged at 1000 *g* for another 5 minutes, the human endometrial stromal cells were plated in T25 flasks. The stromal cells were subsequently cultured in Dulbecco's modified Eagle's/F12 medium (DMEM/F12; HyClone) and supplemented with 20% fetal bovine serum (FBS; HyClone), 100 U/mL penicillin, and 100 mg/mL streptomycin (HyClone) in humidified atmosphere with 5% CO_2_ at 37°C. The purity of isolated stromal cells was >95%, and stromal cells were contaminated by less than 1% of epithelial cells, as determined by diffuse and strong cytoplasmic immunostaining for Vimentin (diluted 1:50; Abcam, Cambridge, UK) and negative cellular staining for E‐cadherin (diluted 1:50; Abcam, Cambridge, UK) in immunocytochemistry.

### 2.5 Hypoxia treatment

After passage 0‐1 when human endometrial stromal cells were nearly confluent, the endometrial stromal cells (4 × 10^5^) were trypsinized and re‐plated in 60 mm culture dishes. To induce hypoxia, cells were cultured in a sealed modular incubator chamber (Thermo Fisher Scientific, Rochester, NY, USA) containing humidified hypoxic air (1% O_2_, 5% CO_2_, 94% N_2_) for the indicated times at 37°C. Control cells were incubated under normoxic conditions (21% O_2_, 5% CO_2_, 37°C) for equivalent periods.

### 2.6 Characterization and identification of isolated human endometrial stromal cells

Immunocytochemistry assay was performed to detect mesenchymal marker vimentin and epithelial marker E‐cadherin. Briefly, isolated human endometrial stromal cells were plated into a 6‐well plate at a density of 2 × 10^4^ cells/well and grown until approximately 60% confluent. The cells were fixed with 4% paraformaldehyde at 4°C for 15 minutes and permeabilized by 0.3% Triton X‐100 for 10 minutes to increase their permeability to antibodies. Non‐specific binding of the antibodies was avoided by blocking with 1% bovine serum albumin (BSA) in PBS for 1 h at room temperature, followed by incubation with primary antibodies of E‐cadherin (diluted 1:50; Abcam, Cambridge, UK) and Vimentin (diluted 1:50; Abcam, Cambridge, UK) overnight at 4°C, and then with horseradish peroxidase‐conjugated secondary antibody (diluted 1:500; Servicebio Biotech, Wuhan, China) for 1 hour at 37°C. The cells were washed with PBS and were stained with Mayer's haematoxylin for nuclei as a counter staining. The cells were observed and photographed by an Eclipse TE2000‐S microscope system (Nikon UK Ltd, Surrey) with Image‐Pro Plus program (Media Cybernetics UK, Berkshire).

### 2.7 RNA extraction and quantitative real time polymerase chain reaction

Total RNA was extracted from collected endometrium tissue biopsies and cultured cells with the use of TRIzol reagent (Takara, Japan) following the manufacturer's instructions. cDNA synthesis was conducted using the PrimeScriptTM RT Master Mix (Takara, Japan) according to the manufacturer's recommendations. The quantitative real time polymerase chain reaction (qRT‐PCR) was performed using the SYBR Premix Ex TaqTM (Takara, Japan) in a Step‐One‐Plus‐TM real time PCR system (Applied Biosystems Inc, Foster City, CA, USA), and the qRT‐PCR results were recorded and analyzed using the instrument's application software. The expression levels of mRNA and lncRNA were normalized with respect to GAPDH and were calculated using the 2–ΔΔCt method. The qRT‐PCR was performed in duplicate in three independent experiments for each experimental condition. The primers sequences used for amplifications are described in Table S2.

### 2.8 Protein extraction and western blot analysis

Collected human endometrium tissues and cultured cells were lysed in radio immunoprecipitation assay (RIPA) buffer (Beyotime Biotechnology, China) containing protease inhibitors (Sigma, USA) and centrifuged at 12 000 *g* at 4°C for 10 minutes. The protein concentration was quantified by means of the BCA assay method with the use of a protein assay kit (Beyotime Biotechnology, China). Thirty micrograms total protein were mixed 1:1 with equal amounts of sample buffer (4% SDS, 10% beta‐mercaptoethanol, and 20% glycerol in 0.125 M Tris, pH 6.8) containing bromophenol blue and heated at 95°C for 10 minutes. The samples were loaded and separated by 12% sodium dodecyl sulfate–polyacrylamide gel electrophoresis gels (PAGE) with running buffer. The proteins separated by SDS‐PAGE were transferred to polyvinylidene difluoride (PVDF) membranes (Immobilon‐P transfer membrane). The membranes were blocked at room temperature for 1 hour with 5% fat‐free milk in Tris‐buffered saline solution (10 mmol/L Tris‐HCl [pH 7.4] and 0.5 mol/L NaCl) containing 0.05% Tween‐20. After three washes for 5 minutes each with the use of TBST, membranes were incubated overnight at 4°C with the following primary antibodies: HIF‐1α (diluted 1:1000; Affinity, USA), LC3 (diluted 1:1000, Abcam, Cambridge, UK), Beclin1 (diluted 1:1000, Abcam, Cambridge, UK), Bax (diluted 1:750; Affinity, USA), Bcl‐2 (diluted 1:1000; Affinity, USA), and GAPDH (diluted 1:1000; Affinity, USA). After this incubation, membranes were washed three times as before and then incubated at room temperature for 1 hour with the HRP‐labelled goat anti‐rabbit secondary antibody (Affinity, USA) at 1:4000 dilution. After three washes for 5 minutes each with the use of TBST, proteins were detected by means of ECL‐Western blot detecting reagent (Millipore, USA) according to the manufacturer's recommendations. The band intensity on western‐blot was quantified by imaging system (Gel Doc 2000; Bio‐Rad, USA) and analysis with Image J software (NIH) (version 1.5, USA) and normalized to GAPDH. Characteristics of antibody used for western blot are listed in Table S3.

### 2.9 Acridine Orange staining

We used AO staining for detection and quantification of acidic vesicular organelles (AVO) in autophagic cells. Acridine orange could be used as an indicator of autophagy, the volume of the cellular acidic compartment was visualized by Acridine orange staining. The cellular acidic compartment volume is increased in autophagy and therefore staining of the acidified autophagosome is used as a reliable autophagy marker.^30^ Briefly, 5 × 10^4^ cells were stained with 1 μg/mL acridine orange (AO) (Sigma‐Aldrich, St. Louis, MO, USA) in PBS and incubated for 15 minutes at 37°C in the dark. After incubation, cells were washed twice with PBS and immediately visualized using an inverted fluorescence microscope (IX51, Olympus, Tokyo, Japan). The autophagy was measured by quantification of the rate of AO positive stained vacuoles in five random fields (a field containing at least 40 cells) for each experimental condition.

### 2.10 Monodansylcadaverine (MDC) staining

Cells were cultured for 24 hours in 12‐well glass‐covered chamber slides at a density of 1 × 10^5^ cells per dish and then cultured under normoxic and hypoxic conditions for the indicated time. Slides were washed with PBS for three times and fixed in 10% formalin solution for 10 minutes. Then cells were exposed to 0.05 m mol L^−1^ MDC (Sigma‐Aldrich, St. Louis, MO, USA) for 15 minutes at 37°C in the dark and visualized using an inverted fluorescence microscope (IX51, Olympus, Tokyo, Japan). The autophagy was measured by quantification of the rate of MDC positive stained vacuoles in five random fields (a field containing at least 40 cells) for each experimental condition.

### 2.11 Transmission electron microscopy (TEM)

Cell samples were fixed with 2% glutaraldehyde‐paraformaldehyde in 0.1 M Na‐phosphate buffer (PB), pH 7.4 for 12 hours at 4°C and washed three times for 30 minutes in 0.1 M PB. Samples were then post‐fixed with 1% OsO4 dissolved in 0.1 M cacodylate buffer (pH 7.4) for 3 hours and dehydrated in an ascending gradual series of ethanol and gradually infiltrated with epoxy resin mixture (812 resin embedding media kit). The samples were sequentially polymerized at 37°C for 12 hours, 45°C for 12 hours, and 60°C for 24 hours. Ultrathin sections (50‐70 nm) were cut by using LKB microtome and mounted on single‐slot copper grids. The sections were subjected to double staining with uranyl acetate and lead citrate and examined using a transmission electron microscope (FEI Tecnai G20, Super twin, Double tilt, LaB6 Gun, USA) at an acceleration voltage of 120 kV.

### 2.12 Autophagy detection using GFP‐ mRFP‐LC3 adenoviral vector

Human endometrial stromal cells were plated in 12‐well plate and allowed to reach 50%‐70% confluence at the time of infection. The tandem GFP‐RFP‐LC3 adenovirus construct were purchased from HanBio Technology Co. Ltd. (HanBio, shanghai, China). Adenoviral was infected into the cells according to the manufacturer's protocol. After being incubated in growth medium with the adenoviruses at a MOI of 100 for 24 hours to ensure the expression of GFP‐ mRFP‐LC3, then the cells were subjected to normoxic and hypoxia conditions for another 24 hours. Autophagy was observed using a laser‐scanning confocal microscope (Olympus America Inc, Center Valley, PA). Autophagic flux was determined by evaluating the number of GFP and mRFP puncta (puncta/cell were counted).

### 2.13 RNA interference

Human lncRNA‐MALAT1 small interfering RNA (siRNA), HIF‐1α siRNA, Beclin1 siRNA, and non‐specific negative control siRNA (si‐NC) were synthesized by RIBOBIO (Guangzhou, Guangdong, China). HIF‐1α overexpression plasmid (pG/CMV/HIF‐1α/IRES/EGFP) and empty vector plasmid were purchased from Gemma Pharmaceutical Technology (Suzhou, Jiangsu, China). Briefly, human endometrial stromal cells (2 x 10^5^ cells/well) were seeded in 6‐well plates and grown to 60%‐80% confluence, and then transfected with 50 n mol L^−1^ siRNA using transfection reagent Lipofectamine 2000 (Invitrogen Life Technologies, USA) according to the manufacturer's instructions. After a 6 hours antibiotic‐free medium incubation, the transfection mixture was removed, and the cells were incubated in DMEM/F‐12 with 20% FBS for another 24 hours, followed by further drug treatments. Sequences for siRNAs used in this study are shown in Table S2.

### 2.14 Cell viability assays

For cell viability assays, human endometrial stromal cells were plated in 96‐well dishes at a density of 2 × 10^4^ cells/well (100 μl). The cells cultured under normoxia and hypoxic condition were treated with 10 m mol L^−1^ 3‐MA or transfected with Beclin1 siRNA for 24 hours. After indicated treatment, 3‐(4,5‐dimethylthiazol‐2‐yl)‐2,5‐diphenyltetrazolium bromide (MTT, 5 mg/ml, Sigma) MTT was added into each well with a final concentration of 0.5 mg/mL, and cells were incubated for 4 h at 37°C in the dark. After incubation, the supernatant was aspirated and formazan crystals were dissolved in dimethyl sulfoxide (DMSO, 150 μl/well) at 37°C for 15 min with gentle agitation. The absorbance per well was monitored at 490 nm using a microplate reader (Bio‐Rad, Philadelphia, PA, USA). The percentage of cell viability was calculated using the following formula: % cell viability = (mean absorbance in test wells)/(mean absorbance in control wells) ×100.

### 2.15 Hoechst staining

The detection of apoptosis was displayed using the Heochst staining 33342 (KeyGen BioTech, Nanjing, China). Briefly, human endometrial stromal cells were seeded in 6‐well plates. The cells cultured under normoxia and hypoxic condition were treated with 10 m mol L^−1^ 3‐MA or transfected with Beclin1 siRNA for 24 hours. After indicated treatment, cells were stained with Hoechst for 15 minutes in the dark and washed by PBS for 5 minutes three times. Cell observation used an inverted fluorescence microscope (IX51, Olympus, Tokyo, Japan).

### 2.16 Statistical analysis

Statistical analyses were performed using Graphpad Prism^®^ statistical analysis software (version 6.01; GraphPad Software Inc., CA, USA). All data presented here are the mean ± standard deviations (SD). The Student's *t* test was used to assess the significance of differences in protein levels of HIF‐1α and LC3 in different type of endometrial tissues. Statistical comparison among multiple groups was performed using one‐way analysis of variance (ANOVA). Differences with *P* values of <0.05 were considered statistically significant. Each experiment was independently repeated at least three times.

## 3 RESULTS

### 3.1 Morphology and identification of primary human endometrial stromal cells

**Figure 1:**
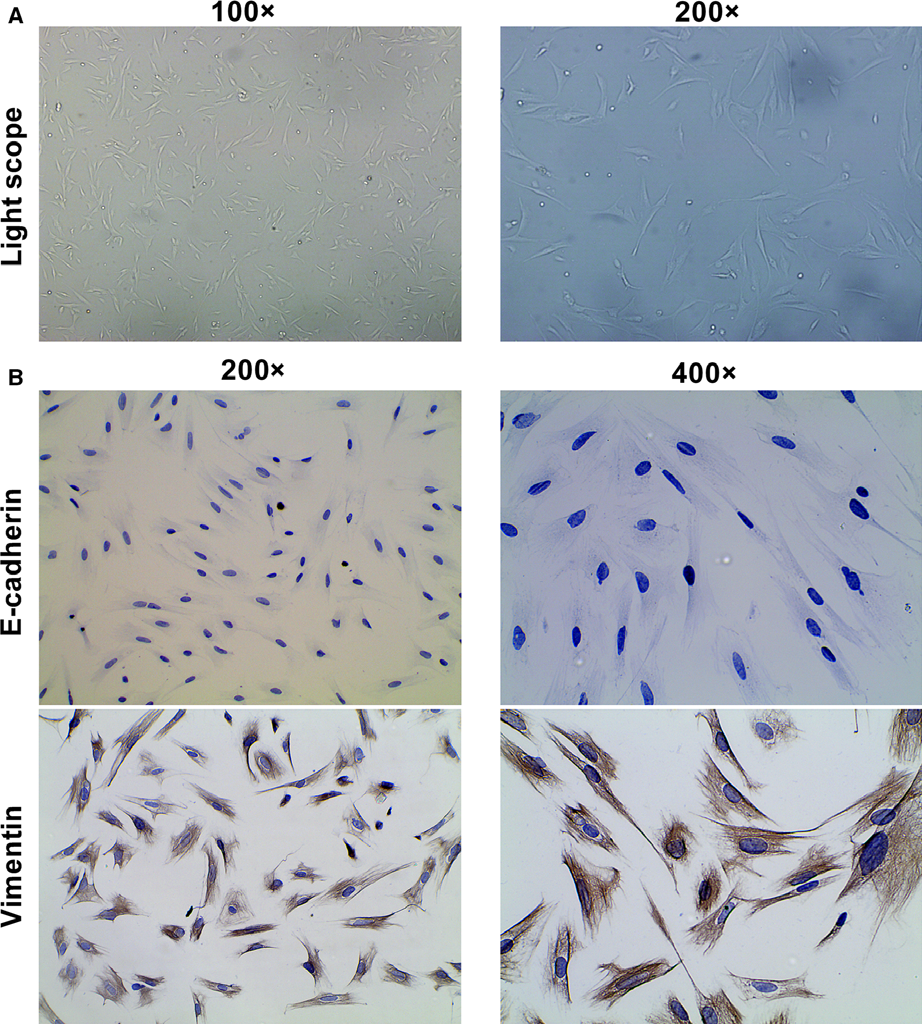
Identification of the primary human endometrial stromal cells. A, Representative of morphology of cultured endometrial stromal cells. Photographs were taken at magnifications of 100× (left panels) and 200× (right panels), respectively. B, Representative of immunocytochemistry staining of E‐cadherin and vimentin protein in endometrial stromal cells. Photographs were taken at magnifications of 200× (left panels) and 400× (right panels), respectively

Vimentin and E‐cadherin are the specific markers of human endometrial stromal cells and human endometrial glandular epithelial cells, respectively. As shown in Figure 1A,B, human endometrial stromal cells displayed long‐spindle and typical fibroblast‐like shapes with positively expressing mesenchymal specific markers Vimentin and negatively expressing epithelial specific marker E‐cadherin. The purity of primary human endometrial stromal cells in isolated cells was >95%.

### 3.2 Expression of MALAT1, HIF‐1α, and LC3 in ectopic endometrium of endometriosis

**Figure 2:**
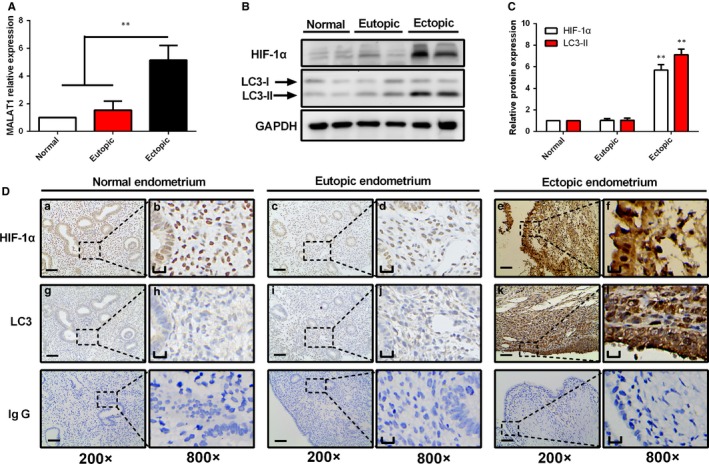
The expression levels of lncRNA‐MALAT1, HIF‐1α, and LC3 in different groups of endometria. A, Significant higher expression of lncRNA‐MALAT1 was found in ectopic endometrium tissues of endometriosis than in normal tissues and eutopic endometrium tissues of endometriosis. (B‐C) Representative Western blots of HIF‐1α and LC3 protein from normal endometrium, eutopic endometrium, and ectopic endometrium. D, Representative immunohistochemical images of HIF‐1α and LC3 protein localization in normal endometrium, eutopic endometrium, and ectopic endometrium. Photographs were taken at magnifications of 200× (left panels) and 800× (right panels), respectively. Left bar = 50 μm, right bar = 12.5 μm. The protein expression levels were quantified by Image J software and normalized to GAPDH protein levels. Quantitative results are expressed as the means ± SD of at least three independent experiments (**P <* *0.01)

To investigate whether lncRNA‐MALAT1, HIF‐1α, and autophagy‐associated marker LC3 play a role in endometriosis, we measured their expression in ovarian endometriosis specimens. Firstly, using qRT‐PCR, we found that lncRNA‐MALAT1 expression was higher in ovarian endometriosis tissues than that in normal and eutopic endometrium with endometriosis (Figure 2A). Then, Western blot assay was performed to detect the expression levels of HIF‐1α and autophagy marker LC3. As shown in Figure 2B,C, the data revealed that increased protein expression of HIF‐1α and total accumulation of LC3‐II were present in ovarian endometriosis samples compared with normal and eutopic endometrium from women with endometriosis. Afterwards we further investigated the location and protein levels of HIF‐1α and LC3 by immunohistochemical staining. HIF‐1α was pre‐dominantly localized in the nuclei of epithelial and stromal cells; however, LC3 was expressed within the cytoplasm of both cell types. The expression levels of HIF‐1α and LC3 in the ectopic endometrium were significantly greater than those in normal endometrium and eutopic endometrium from women with endometriosis (Figure 2D). Taken together, the data provided evidence that the autophagy process occurred during ovarian endometriosis, and elevated expression of HIF‐1α and lncRNA‐MALAT1 may be involved in the pathological process.

### 3.3 Hypoxia induced activation of autophagy in human endometrial stromal cells

**Figure 3:**
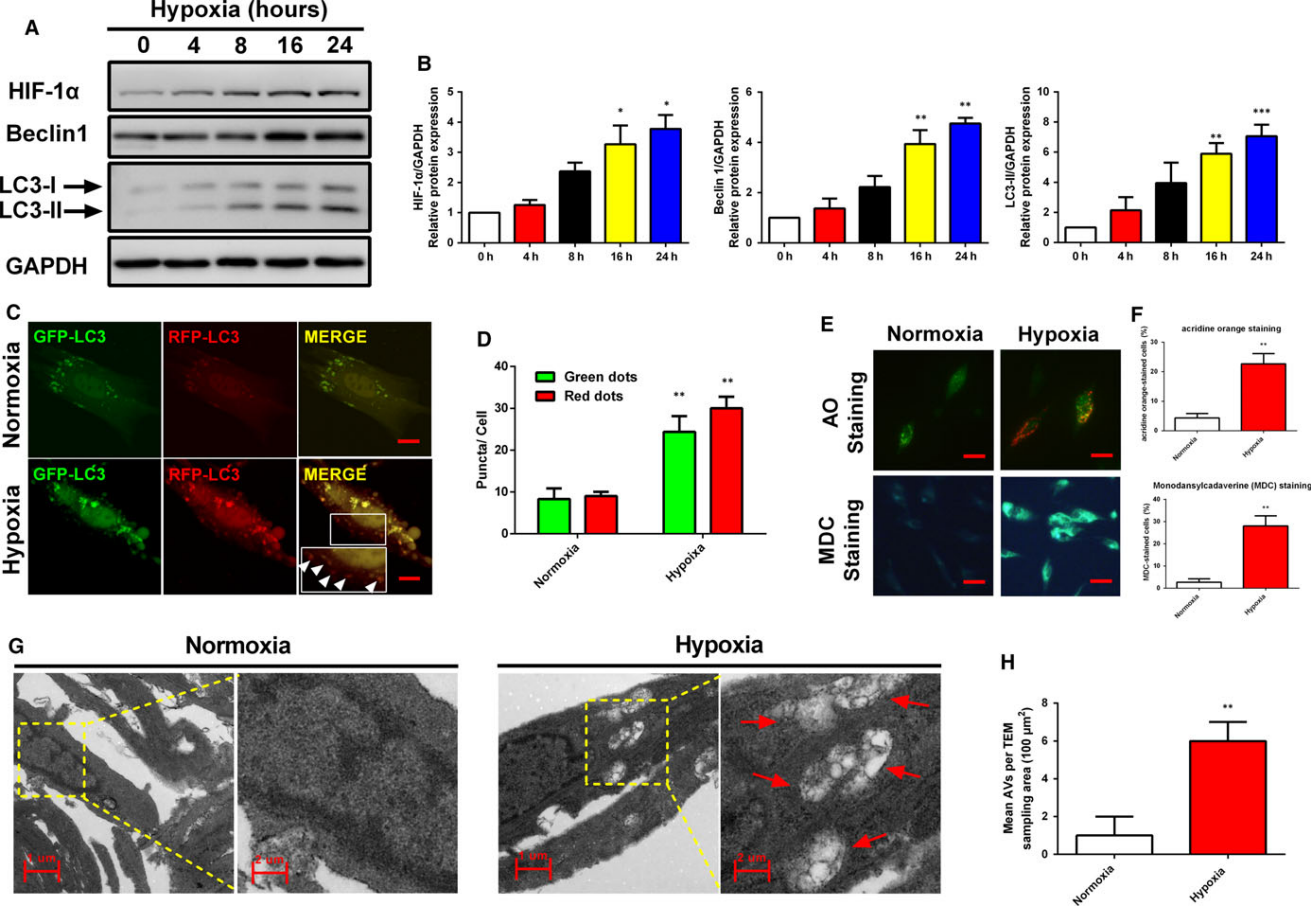
Hypoxia induced autophagy in human endometrial stromal cells. (A‐B) Human endometrial stromal cells were incubated under hypoxic conditions for 0, 4, 8, 16, and 24 h and then analysed by Western blot to determine the expression levels of HIF‐1α,Beclin1, and LC3. **(**C‐D) Representative images showing LC3 staining in different groups of human endometrial stromal cells infected with GFP‐RFP‐LC3 adenovirus for 24 h (Scale bar: 5 μm). Quantification of mean red and green fluorescent puncta of at least 10 cells per condition is shown. (E‐F) Representative images of human endometrial stromal cells treated with normoxia and hypoxia for 24 h, respectively, and then cells were stained with acridine orange and MDC (Scale bar: 20 μm). The percentage of cells stained for acridine orange or MDC were quantified. (G‐H) Representative images of human endometrial stromal cells incubated with normoxia (left panel) and hypoxia (right panel) for 24 h, and then were analysed by Transmission electron microscopy (TEM). Autophagical vacuoles containing organelle remnants were highlighted by red arrows. The protein expression levels were quantified by Image J software and normalized to GAPDH protein levels. Quantitative results are expressed as the means ± SD of at least three independent experiments (*P <* *0.05; **P <* *0.01; ***P <* *0.001)

To explore whether autophagy activation was triggered by hypoxia stress in human endometrial stromal cells, we firstly examined the protein expression levels of HIF‐1α, Beclin1, and LC3‐II, which are considered reliable indicators of autophagy. As shown in Figure 3A,B, there was a gradual increase in the protein expression levels of HIF‐1α, Beclin1, and LC3B‐II under hypoxia. To further confirm that hypoxia can induce complete autophagic flux, we utilized the tandem GFP‐RFP‐LC3 adenovirus construct to further confirm autophagy induction. This is a convenient method for monitoring autophagic flux base on the pH sensitivity difference between autolysosomes and autophagosomes and the pH sensitivity differences exhibited by green fluorescent protein (GFP) and red fluorescent protein (mRFP) to monitor progression from autophagosome to autolysosome.^31^ The green fluorescence quenches when autophagosome fuses with a lysosome to form autolysosomes, at which time only red fluorescence is detected. As shown in Figure 3C,D, more red puncta were presented in hypoxia treated human endometrial stromal cells than in control under normoxia condition. These results further confirmed the induction of autolysosome formation, indicating that hypoxia mediated autophagy flux in human endometrial stromal cells. Furthermore, acridine orange (AO) and monodansylcadaverine (MDC) staining were performed to observe acidic vesicular organelles (AVOs), which accurately indicates autophagic activity. As shown in Figure 3E,F, compared with normoxia group, human endometrial stromal cells treated with hypoxia showed more accumulation of autophagic vacuoles. Autophagosomes were first monitored by transmission electron microscopy (TEM), and TEM remains one of the most reliable methods for the observation of autophagy and quantification of autophagic accumulation.^32^ By TEM analysis, we determined that hypoxia treatment increased the number of cytoplasmic autophagosomes in human endometrial stromal cells (Figure 3G,H). In summary, these findings indicated that hypoxia induces autophagy in human endometrial stromal cells.

### 3.4 LncRNA‐MALAT1 is induced by hypoxia and regulated by HIF‐1α in human endometrial stromal cells

**Figure 4:**
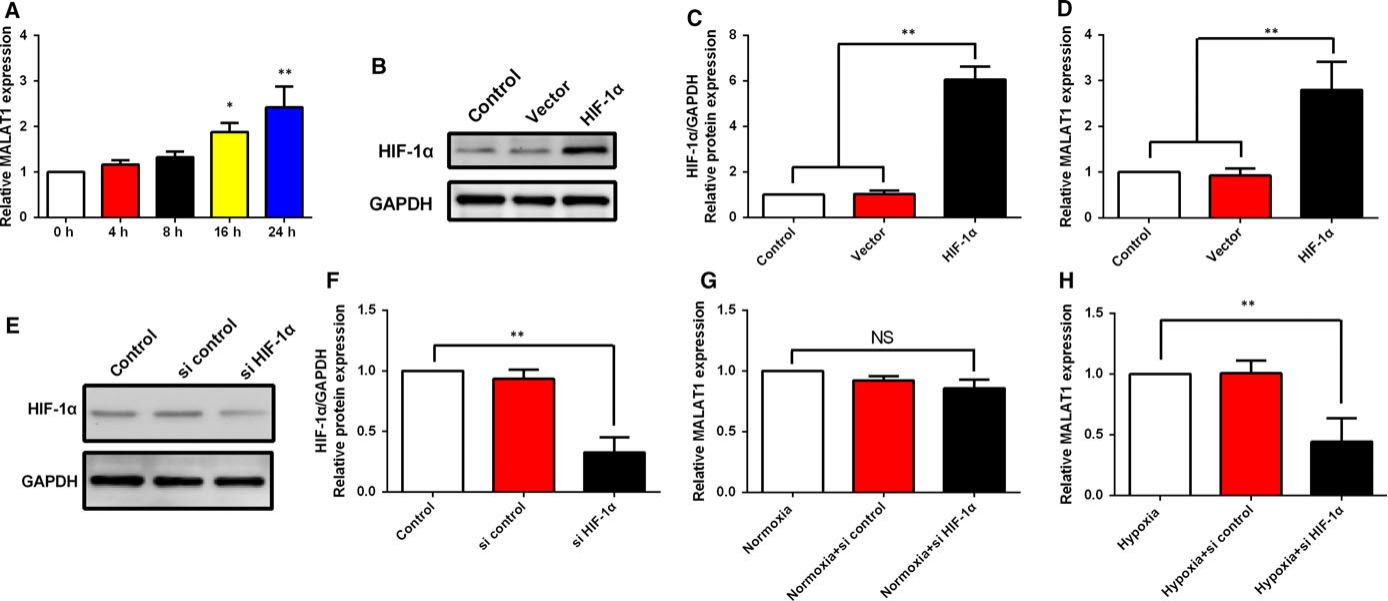
lncRNA‐MALAT1 is induced by hypoxia and regulated by HIF‐1α in human endometrial stromal cells. A, Human endometrial stromal cells were incubated under hypoxic conditions for 0, 4, 8, 16, and 24 h. lncRNA‐MALAT1 levels was examined by qRT‐PCR. (B‐C) Human endometrial stromal cells were transfected with empty vector or HIF‐1α expression plasmid before culture under normoxia for 24 h. HIF‐1α protein expression levels was examined by Western blot. D, Human endometrial stromal cells were transfected with empty vector or HIF‐1α expression plasmid before culture under normoxia for 24 h, and lncRNA‐MALAT1 levels was examined by qRT‐PCR. (E‐F) Human endometrial stromal cells were transfected with control siRNA or HIF‐1α specific siRNA before culture under normoxia for 24 h. HIF‐1α protein expression levels was examined by Western blot. (G‐H) Human endometrial stromal cells were transfected with control siRNA or HIF‐1α specific siRNA before culture under normoxia or hypoxia conditions for 24 h, and lncRNA‐MALAT1 levels were examined by qRT‐PCR. The protein expression levels were quantified by Image J software and normalized to GAPDH protein levels. Quantitative results are expressed as the means ± SD of at least three independent experiments (*P <* *0.05; **P <* *0.01)

In order to reveal the mechanism underlying the up‐regulation of lncRNA‐MALAT1 in human ovarian endometriosis tissues, human endometrial stromal cells were treated with hypoxia for various time periods. As shown in Figure 4A, qPCR analysis revealed a time‐dependent increase of lncRNA‐MALAT1 in human endometrial stromal cells. As HIF‐1 is the master regulator of the cellular response to hypoxia, it is considered to transcriptionally regulate the expression of lncRNA‐MALAT1. To investigate the mechanism underlying lncRNA‐MALAT1 up‐regulation during hypoxia, a HIF‐1α empty vector and overexpression plasmid was designed and transfected into human endometrial stromal cells. Western blot analysis was performed and revealed that HIF‐1α was overexpressed successfully, together with the increase of lncRNA‐MALAT1 expression level (Figure 4B‐D). Furthermore, negative control siRNA and HIF‐1α‐specific‐siRNA sequences were designed and transfected into human endometrial stromal cells to further evaluate the effects of HIF‐1α on lncRNA‐MALAT1 expression. The HIF‐1α‐siRNA obviously inhibited the expression of HIF‐1α (Figure 4E‐F). Knock down of HIF‐1α did not affect lncRNA‐MALAT1 expression under normoxia condition, whereas lncRNA‐MALAT1 was markedly down‐regulated under hypoxia condition (Figure 4G‐H). Taken together, these data implicated that lncRNA‐MALAT1 was up‐regulated by HIF‐1α under hypoxia condition.

### 3.5 Knockdown of lncRNA‐MALAT1 interferes with hypoxia‐induced autophagy in human endometrial stromal cells

**Figure 5:**
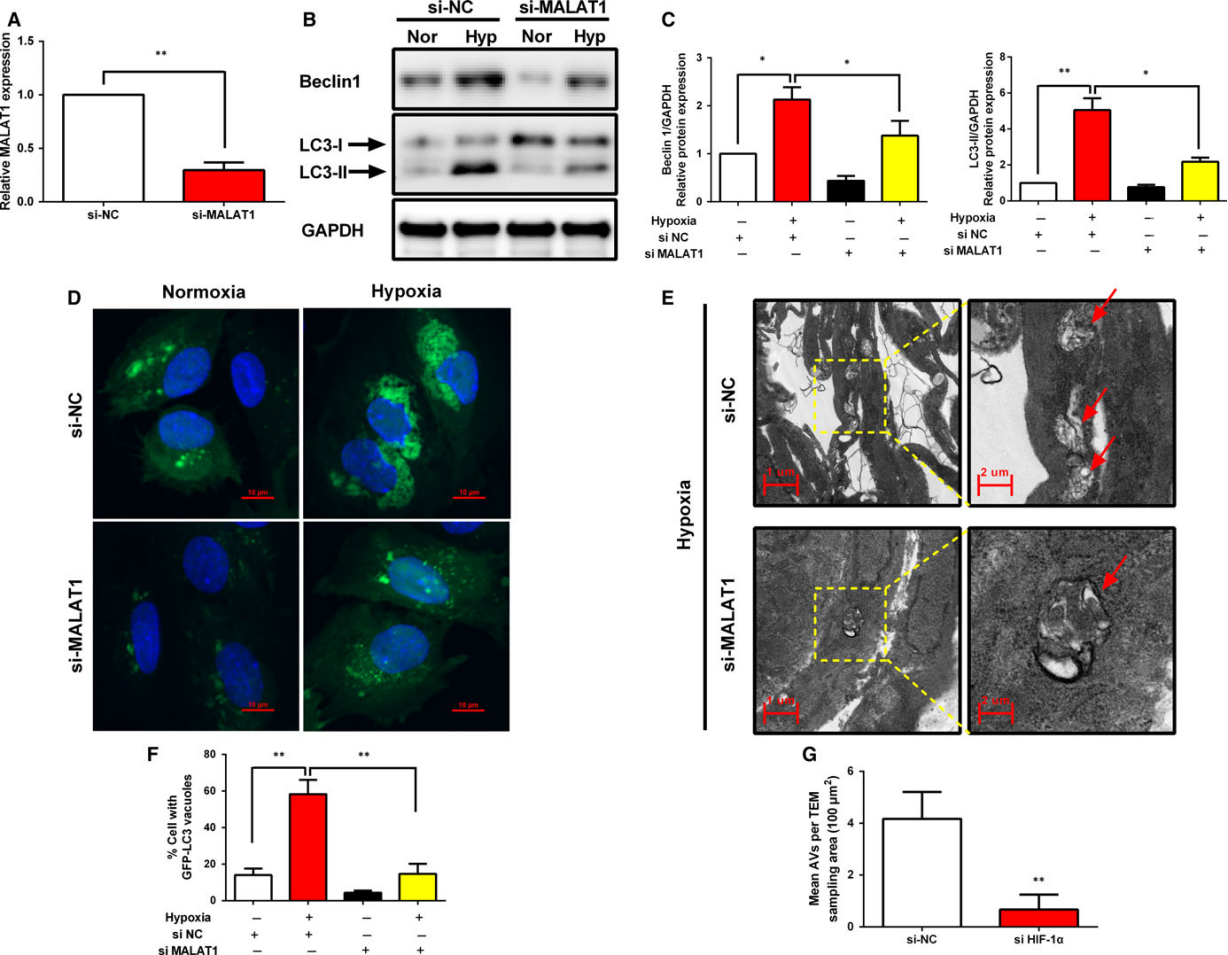
Knockdown of lncRNA‐MALAT1 interferes with hypoxia‐induced autophagy in human endometrial stromal cells. A, qRT‐PCR was used to examine the knockdown efficiency of lncRNA‐MALAT1 siRNA. (B‐C) Representative Western blots of Beclin1 and LC3 protein in human endometrial stromal cells transfected with negative control siRNA or lncRNA‐MALAT1 specific siRNA in the presence or absence of hypoxia for 24 h. D, Representative immunofluorescence image of GFP‐LC3 puncta in human endometrial stromal cells transfected with negative control siRNA or lncRNA‐MALAT1 specific siRNA under hypoxia condition for 24 h. Photographs were taken at magnifications of 1600×. E, Representative images of human endometrial stromal cells transfected with negative control siRNA or lncRNA‐MALAT1 specific siRNA under hypoxia condition for 24 h, and then were analysed by Transmission electron microscopy (TEM). Autophagical vacuoles containing organelle remnants were highlighted by red arrows. The protein expression levels were quantified by Image J software and normalized to GAPDH protein levels. The data are presented as the means ± SD of three independent experiments (*P <* *0.05; **P <* *0.01)

To investigate the detailed role of lncRNA‐MALAT1 in the regulation of hypoxia induced autophagy in human endometrial stromal cells, we first constructed a siRNA specifically targeting lncRNA‐MALAT1. The data revealed that lncRNA‐MALAT1 siRNA (si‐MALAT1) transfection successfully down‐regulated lncRNA‐MALAT1 expression level (Figure 5A) when compared with a negative control that the cells were treated with, siRNA (si‐NC). Additionally, Western blot was used to investigate the changes of autophagy in si‐MALAT1‐transfected human endometrial stromal cells with or without hypoxia treatment. Western blot results showed that compared with human endometrial stromal cells transfected with si‐NC, lncRNA‐MALAT1 knockdown significantly reduced Beclin1 and LC3‐II protein levels under hypoxia condition, indicating that knockdown of lncRNA‐MALAT1 could attenuate hypoxia‐induced autophagy (Figure 5B‐C). Fluorescence microscopy showed that under hypoxia conditions, levels of GFP‐LC3 punctate formation in human endometrial stromal cells transfected with si‐NC were higher than that in normoxia condition. However, upon hypoxia treated human endometrial stromal cells transfected with MALAT1 siRNA showed a significant decrease in the numbers of GFP‐LC3 punctate (Figure 5D,F). In addition, TEM analysis revealed that autophagosomes accumulation was significantly decreased in human endometrial stromal cells transfected with MALAT1 siRNA compared to that of negative control group under hypoxia condition (Figure 5E,G). These findings suggest the crucial importance of up‐regulated lncRNA‐MALAT1 expression in regulating hypoxia triggered autophagy, and demonstrate a potential role for lncRNA‐MALAT1 in promoting autophagy activation in human endometrial stromal cells. Taken together, this data showed that lncRNA‐MALAT1 contributed to hypoxia triggered autophagy activation in human endometrial stromal cells.

### 3.6 Autophagy inhibition by 3‐methyladenosine (3‐MA) or si Beclin1 promotes human endometrial stromal cells apoptosis under hypoxia condition

**Figure 6:**
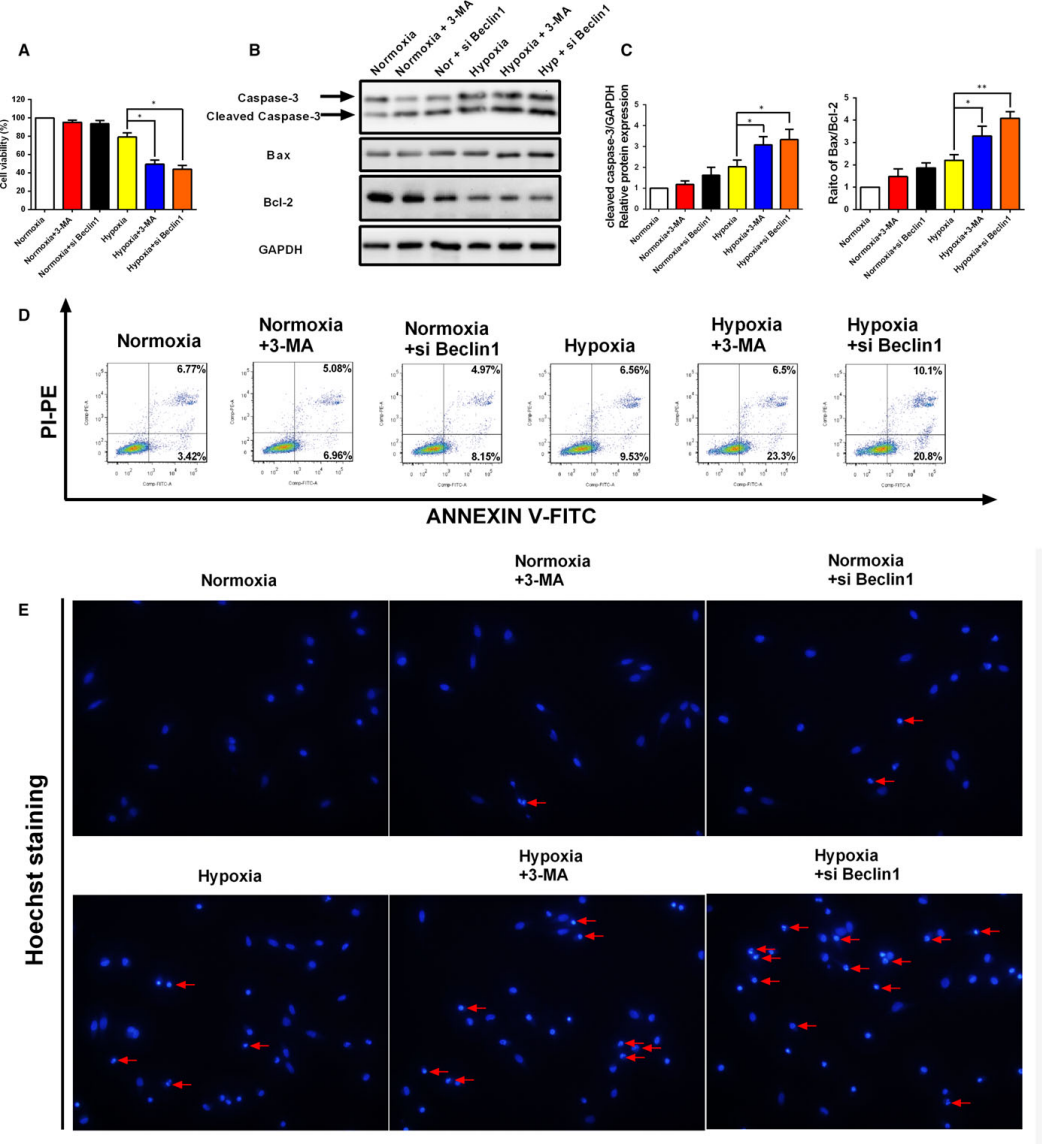
Inhibition of autophagy by 3‐MA and si Beclin1 promotes apoptosis of human endometrial stromal cells under hypoxia condition. A, Representative images of the cell viability in human endometrial stromal cells after treated with 3‐MA and si Beclin1 with or without the presence of hypoxia. (B‐C) Representative Western blot of cleaved caspase‐3 and Bax/Bcl2 ratio in human endometrial stromal cells after treated with 3‐MA and si Beclin1 with or without the presence of hypoxia. D, Representative flow cytometry images of cell apoptosis in human endometrial stromal cells after treated with 3‐MA and si Beclin1 with or without the presence of hypoxia. E, Representative fluorescence images of human endometrial stromal cells stained with Hoechst 33342 fluorescent dye. Human endometrial stromal cells were treated with 3‐MA and si Beclin1 with or without the presence of hypoxia. Photographs were taken at magnifications of 200×. The protein expression levels were quantified by Image J software and normalized to GAPDH protein levels. The data are presented as the means ± SD of three independent experiments (*P <* *0.05; **P <* *0.01)

Next, we determined whether autophagy induction contributes to the aberrant survival of human endometrial stromal cells under hypoxia condition. Firstly, 3‐(4,5‐cimethylthiazol‐2‐yl)‐2,5‐diphenyl tetrazolium bromide (MTT) assay revealed that autophagy inhibition by 3‐MA and Beclin‐1 knockdown in human endometrial stromal cells caused significant decreases in cell viability under hypoxia condition (Figure 6A). Western blot results in Figure 6B,C revealed that the expression of cleaved caspase‐3 and Bax/Bcl‐2 ratio significantly increased in human endometrial stromal cells treated with 3‐methyladenosine (3‐MA) and si Beclin1 under hypoxia condition compared with normoxia condition. To further confirm these results, we assayed cell apoptosis with flow cytometry. The data, as indicated by the annexin V‐FITC assay, showed that only 2.7% of the cells underwent apoptosis under normoxia conditions, while approximately 10.4% of the cells under the hypoxia condition were apoptotic (Figure 6D). However, inhibition of autophagy by using 3‐MA or Beclin1 siRNA was found to distinctly increased cell apoptosis under the hypoxia condition, which indicates the inhibition of the protective effect of autophagy. Hoechst 33342 staining further revealed that inhibition of autophagy significantly increased the number of DNA damaging and nuclear fragmentation in human endometrial stromal cells (Figure 6E). Collectively, these findings indicated that autophagy induction contributes to survival of human endometrial stromal cells under hypoxia conditions.

## 4 DISCUSSION

To the best of our knowledge, this is the first report on the effects of LncRNA‐MALAT1 on autophagy which was induced by hypoxia in human endometrial stromal cells. In the present study, we found that the expression of lncRNA‐MALAT1 was remarkably up‐regulated and positively correlated with the expression of HIF‐1α and autophagy marker LC3 in ovarian endometriosis tissue samples. Furthermore, hypoxia markedly up‐regulated the levels of lncRNA‐MALAT1 in a HIF‐1α dependent manner. Elevation of lncRNA‐MALAT1 by hypoxia causes an activation of pro‐survival autophagy pathway, which results in reduced cell apoptosis in endometriosis.

Endometriosis is considered to be a polygenically inherited disorder with a complex, multi‐factorial aetiology.^33^ Many different theories relating to the pathogenesis of endometriosis have been put forward and to date they all remain to be conclusively confirmed. Sampson's transplantation and implantation theory, which is the most widely accepted assumption, indicates that endometriosis occurs due to the reflux of endometrial debris by retrograde menstruation.^34^ However, because retrograde menstruation occurs in most women but only 5%‐10% develop endometriosis, other factors such as peritoneal hypoxia microenvironment must contribute to the development of this disease.^8^, ^35^ According to Sampson's theory, when shed endometrial cells or debris retrogrades to the peritoneal cavity, the first stress faced is the local altered hypoxic microenvironment. A growing body of evidence suggests that peritoneal local hypoxia play an important role in endometriosis development and progression and the expression of hypoxia‐inducible factor‐1alpha (HIF‐1α) was increased significantly in the development of endometriosis.^5^, ^6^, ^36^

Although the etiology of endometriosis remains largely unknown, a considerable amount of evidence supports that dysregulation of LncRNAs may play a role in the pathological processes of endometriosis.^20^, ^21^ Among them, lncRNA‐MALAT1 is an abundant and highly conserved lncRNA which is often overexpressed in various types of cancers, associating with proliferation, metastasis, and apoptosis.^37^ In this study, by thorough examination of ovarian endometriosis samples, we found that levels of lncRNA‐MALAT1 were significantly overexpressed in ovarian endometriosis samples, compared with normal and eutopic endometrium of endometriosis. This result is consistent with the previous findings of Liang et al, who found that the expression level of lncRNA‐MALAT1 was significantly higher in ectopic endometrium tissue of endometriosis when compared with the paired normal endometrial tissue from the same patient.^28^ More importantly, previous studies reported that hypoxia provides an important driving factor in the expression pattern of various lncRNAs including MALAT1. For example, Sallé‐Lefort et al demonstrated lncRNA‐MALAT1 expression is regulated in hypoxic conditions by a CaMKK/AMPK/HIF‐1α axis.^38^ In another study, Brock et al found that the levels of lncRNA‐MALAT1 were significantly increased in hypoxic human pulmonary artery smooth muscle.^23^ However, whether lncRNA‐MALAT1 is also involved in the hypoxic endometriosis remains uncharacterized. Herein we provide compelling evidence to demonstrate that elevated HIF‐1α expression in ectopic endometriotic tissue is correlated with increased lncRNA‐MALAT1 expression. In addition, lncRNA‐MALAT1 was induced by hypoxia at a time‐dependent manner and is regulated by HIF‐1 α signalling in human endometrial stromal cells. Taking these data together, we demonstrate that hypoxia can up‐regulate endometrial cell lncRNA‐MALAT1 level in a HIF‐1α dependent manner.

Autophagy, as a degradation pathway essential for energy and cellular homoeostasis, serves mainly as a protective mechanism that might prevent cell death under stressful circumstances like hypoxia. Autophagy is triggered by the formation of autophagosomes, relying on the microtubule‐associated protein 1 light chain 3 (LC3) protein and protein‐protein conjugation systems, thus LC3 is always used as a marker for autophagy, and the conversion from LC3‐I to LC3‐II is associated with autophagosome formation. LC3‐II is the first mammalian protein identified that specifically associates with autophagosome membranes. The amount of LC3‐II is correlated with the extent of autophagosome formation, so LC3‐II is widely used for detecting autophagy levels through measuring the ratio of LC3‐ II/Inner control.^39^ Recent research indicated that up‐regulated autophagy was observed in ovarian endometriomas and this process possibly contributing to the survival of endometriotic cells in ectopic sites and to lesion maintenance.^17^ In this study, we first proved that the levels of LC3 was elevated in ectopic endometrium tissues of endometriosis. Our data further demonstrated that autophagy activity was induced by hypoxia at a time‐dependent manner in human endometrial stromal cells. Taking these data together, we demonstrate that hypoxia can up‐regulate endometrial cell autophagy level.

As we described above, both lncRNA‐MALAT1 and autophagy could be up‐regulated by hypoxia stress. Yet the relationship between lncRNA‐MALAT1 and autophagy under hypoxia conditions in the context of endometriosis is still unclear. In recent years, increasing research has emerged to explore the inter‐play between lncRNA‐MALAT1 and autophagy. For example, Li et al showed lncRNA‐MALAT1 served as a potent autophagy inducer protecting brain microvascular endothelial cells against oxygen‐glucose deprivation/reoxygenation‐induced injure.^27^ Moreover, lncRNA‐MALAT1 enhances aggressive pancreatic cancer cells proliferation and metastasis via the up‐regulation of autophagy^40^ and promotes retinoblastoma progression via modulating autophagy.^26^, ^41^ These findings tempted us to speculate that lncRNA‐MALAT1 could also function by regulating autophagy under hypoxia condition in endometriosis. In the present study, we found that silencing lncRNA‐MALAT1 suppresses hypoxia induced autophagy in human endometrial stromal cells. To the best of our knowledge, this is the first report to show the regulation of autophagy by MALAT1 under hypoxia environment in endometriosis.

Accumulating evidences have demonstrated that the endometrial tissues from females with and without endometriosis have fundamental differences in cell survival/apoptosis potentiality.^42^, ^43^ Cells from endometriotic tissues lack the appropriate mechanism of programmed cell death and surviving to establish endometriotic implantation on unfavourable ectopic locations. Moreover, hypoxia induced autophagy has been considered as one cytoprotective mechanism.^44^ However, several aspects of the biological role of autophagy are still unclear, and the relationship between autophagy and apoptosis under hypoxia conditions, particularly in the context of endometriosis, has yet to be thoroughly explored. Recently, Xu et al reported that hypoxia up‐regulates the expression of mirR‐210, which further promotes cell survival and autophagy of endometriotic cells.^45^ But the role of autophagy on apoptosis was not carefully investigated in their study. Our current research has further provided evidence to support a cell protective role for autophagy during hypoxia stress. Here we demonstrated that in cultured human endometrial stromal cells, inhibition of autophagy by either 3‐MA or siRNA knockdown of Beclin‐1 enhanced apoptosis during hypoxic incubation. However, for the reason that autophagy has been ascribed both cytoprotective and pro‐apoptotic functions, and there have been controversial results regarding the role of autophagy in cell death in endometriosis. Choi and coworkers reported that autophagy induction by mTOR inhibition rapamacin could contribute to abnormal apoptosis in endometriotic cell.^46^ In addition, dienogest promotes endometriotic cell apoptosis via the induction of autophagy, suggesting a pro‐apoptotic role for dienogest mediated induction of autophagy in endometriotic cells.^47^ This discrepancy underscores the dichotomous nature of autophagy that could be either protective or detrimental depending upon the different cell state, genetic factors and/or onstage of disease progression. Thus, it needs further studies to clarify the detailed mechanism (s) by which autophagy contributes to cell survival under hypoxia conditions.

However, there are several limitations of our study that must be taken into account. First of all, endometrium samples were only collected during the proliferative phase and the sample size was relatively small, which may limit the interpretable power of the current results. Thus, in future studies, the larger sample sizes collected from the entire menstrual cycle should be used. Second, the exact molecular mechanisms underlying lncRNA‐MALAT1 mediated autophagy in human endometrial stromal cells remains to be established. Thus, future research is needed to gain deeper insight into these questions.

**Figure 7:**
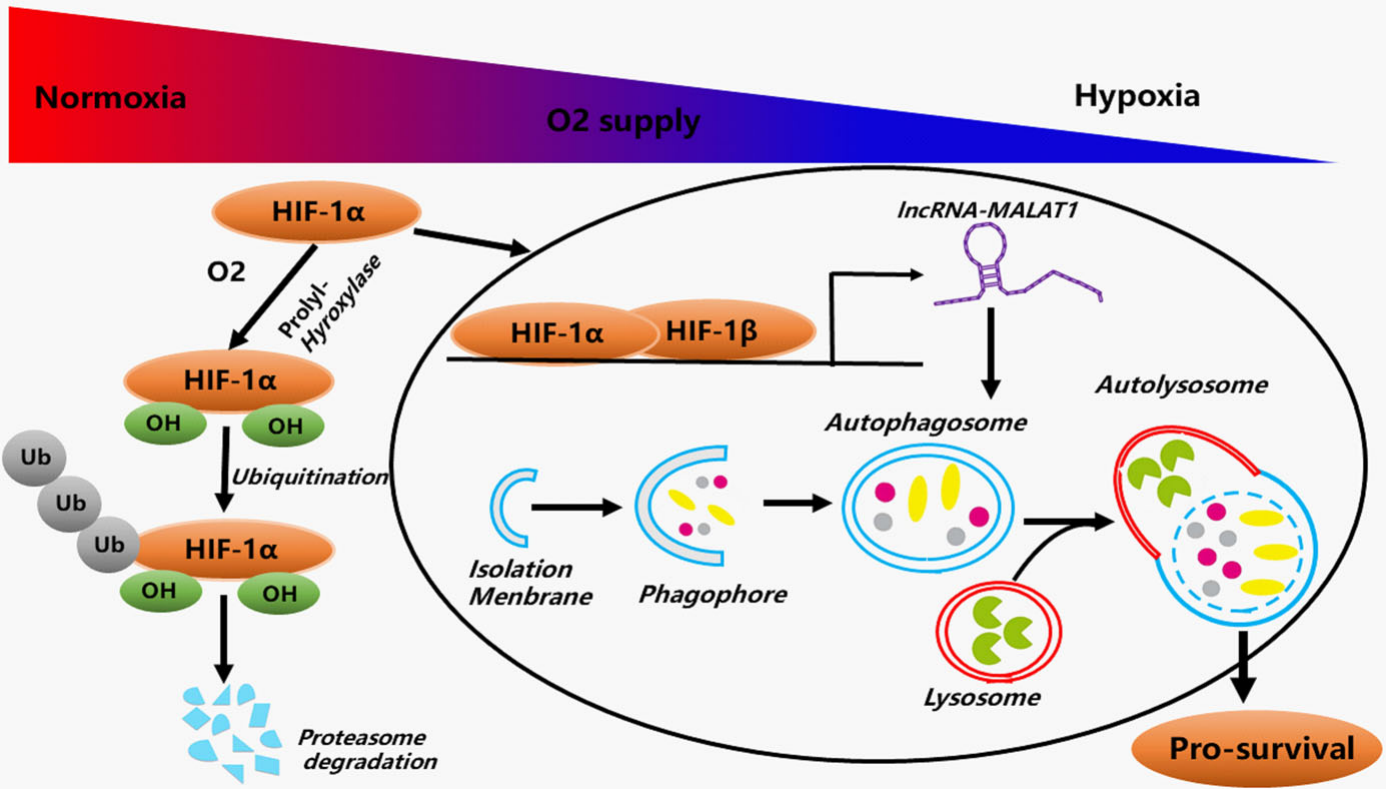
Schematic diagram of the functions of lncRNA‐MALAT1 during hypoxia induced pro‐survival autophagy in human endometrial stromal cells. In normoxia condition, HIF‐1α protein is rapidly degraded by the ubiquitin‐proteasome system. In hypoxia condition, HIF‐1α is stabilized and translocated into the nucleus by importin α/β and dimerizes with HIF‐1β. The HIF heterodimer affects transcription of downstream target lncRNA‐MALAT1, which promotes the induction of pro‐survival autophagy

In summary, to the best of our knowledge, this is the first report showing that lncRNA‐MALAT1 contributes to hypoxia triggered protective autophagy, which is crucial for cell survival in endometriosis (Figure 7). Altogether, these findings illustrate a new function of lncRNA‐MALAT1 and the “hypoxia/lncRNA‐MALAT1/protective autophagy” pathway may serve as a novel target for developing potential therapeutic targets for the treatment of endometriosis.

## CONFLICT OF INTERESTS

The authors declare that there is no conflict of interest that could be perceived as prejudicing the impartiality of the research reported.

## AUTHOR CONTRIBUTIONS

YL, XX, and HL conceived and designed the study. HL performed the cell line experiments. ZZ provided expert advice. All authors analysed the results. HL wrote the manuscript and ZZ, WX, and LZ reviewed the manuscript. All authors read and approved the final manuscript.
