# Supplementary material for: Long non‐coding RNA MALAT1 mediates hypoxia‐induced pro‐survival autophagy of endometrial stromal cells in endometriosis
Source: J Cell Mol Med. 2018 Oct 15;23(1):439–52. doi: 10.1111/jcmm.13947 (PMC6307811; doi:10.1111/jcmm.13947)
Supplement: Supplementary file 1 [file JCMM-23-439-s001.docx]

**Supplemental Table 1.** **Clinical characteristics of patients.**

| Number of cases  *Age  Menstrual cycle phase  #rASRM stage  III  IV | **Samples used for immunohistochemistry**  **Normal Eutopic Ectopic**  **Endometrium Endometrium Endometrium** | | | **Samples used for primary cell cultures** |
| --- | --- | --- | --- | --- |
|  | 30  34.3±2.1  Proliferative  -  - | 30  32.4±3.2  Proliferative  11  19 | 30  36.3±2.5  Proliferative  16  14 | 82  34.2±2.7  Proliferative  -  - |

*Median (range).

#Revised American Society for Reproductive Medicine classification (rASRM: American Society for Reproductive Medicine, 1997).

**Supplemental Table 2. Information for siRNA sequences**

Sequence tags Sequences (5’-3’)

MALAT1 siRNA Sense GGCAAUGUUUUACACUAUUTT

Antisense AAUAGUGUAAAACAUUGCCTA

HIF-1α siRNA Sense CAGUUACGAUUGUGAAGUUAA

Antisense AACUUCACAAUCGUAACUGGU

Beclin1 siRNA Sense CGGGAAUACAGUGAAUUUATT

Antisense UAAAUUCACUGUAUUCCCGTT

negative control siRNA Sense UUCUCCGAACGUGUCACGUTT

Antisense ACGUGACACGUUCGGAGAATT

**Supplemental Table 3. Primer sequences for qRT-PCR primers**

Gene primier sequence (5’-3’)

MALAT1 Forward GCCACTTCTCAACCGTCCCT

Reverse AACACCTCACAAAACCCCCG

**Supplemental Table 4. Commercial sources and characteristics of antibodies used.**

Antibody Dilution Isotype Product Num Location

IHC WB /Manufacture

HIF-1α 1:150 1:1000 Rabbit IgG (Polyclonal) AF1009 / Affinity U.S.

LC3 1:100 1:1000 Rabbit IgG (Polyclonal) ab51520 /Abcam U.K.

Beclin1 None 1:1000 Rabbit IgG (Polyclonal) ab62557/Abcam U.K.

Caspase-3 None 1:500 Rabbit IgG (Polyclonal) ab32351/Abcam U.K.

Bax None 1:1000 Rabbit IgG (Polyclonal) ab32503/Abcam U.K. Bcl-2 None 1:1000 Rabbit IgG (Polyclonal) ab32124/Abcam U.K.

GAPDH None 1:1000 Rabbit IgG (Polyclonal) AF0911 / Affinity U.S.

**Supplemental Table 5. Immunostaining score of HIF-1α and LC3 protein in normal endometrium, eutopic endometrium and ectopic endometrium of endometriosis.**

|  |  |  |
| --- | --- | --- |
|  | **Immunostaining score (mean±SD)** | |
|  | **HIF-1α** | **LC3** |
| Normal endometrium(N), n=30 | 3.526±1.172 | 2.342±2.264 |
| Eutopic endometrium(U), n=30 | 4.227±2.391 | 1.751±1.836 |
| Ectopic endometrium(E=10), n=30 | 8.441±2.527 | 7.639±3.247 |
| Statistical analysis |  |  |
| N versus U | *P* > 0.05 | *P* > 0.05 |
| N versus E | **P* < 0.05 | **P* < 0.05 |
| U versus E | **P* < 0.05 | ***P* < 0.01 |
|  |  |  |

**p* ＜ 0.05, ***p＜* 0.01

All data are expressed as mean±SD.

Statistical significance (one-way ANOVA analysis).
